# Mild Phenotype of Arthrogryposis, Renal Dysfunction, and Cholestasis Syndrome 1 Caused by a Novel *VPS33B* Variant

**DOI:** 10.3389/fgene.2022.796759

**Published:** 2022-02-25

**Authors:** Natália Duarte Linhares, Eleonora Druve Tavares Fagundes, Alexandre Rodrigues Ferreira, Thaís Costa Nascentes Queiroz, Luiz Roberto da Silva, Sergio D. J. Pena

**Affiliations:** ^1^ Laboratório de Genômica Clínica, Faculdade de Medicina, Universidade Federal de Minas Gerais, Belo Horizonte, Brazil; ^2^ Departamento de Bioquímica e Imunologia, Instituto de Ciências Biológicas, Universidade Federal de Minas Gerais, Belo Horizonte, Brazil; ^3^ Departamento de Pediatria, Faculdade de Medicina, Universidade Federal de Minas Gerais, Belo Horizonte, Brazil; ^4^ Hospital das Clínicas, Universidade Federal de Minas Gerais, Belo Horizonte, Brazil; ^5^ Hospital de Clínicas—EBSERH, Universidade Federal de Uberlândia, Uberlândia, Brazil; ^6^ Laboratório Gene—Núcleo de Genética Médica, Belo Horizonte, Brazil

**Keywords:** whole exome sequencing, VPS33B gene, cholestasis, arthrogryposis, renal dysfunction

## Abstract

The arthrogryposis, renal dysfunction, and cholestasis syndrome (ARCS) is an autosomal recessive multisystem disease caused by variants in *VPS33B* or *VIPAS39*. The classical presentation includes congenital joint contractures, renal tubular dysfunction, cholestasis, and early death. Additional features include ichthyosis, central nervous system malformations, platelet dysfunction, and severe failure to thrive. We studied three patients with cholestasis, increased aminotransferases, normal gamma-glutamyl transferase, and developmental and language delay. Whole exome sequencing analysis identified *VPS33B* variants in all patients: patients 1 and 2 presented a novel homozygous variant at position c.1148T>A. p.(Ile383Asn), and patient 3 was compound heterozygous for the same c.1148T>A. variant, in addition to the c.940-2A>G. variant. ARCS is compatible with the symptomatology presented by the studied patients. However, most patients that have been described in the literature with ARCS had severe failure to thrive and died in the first 6 months of life. The three patients studied here have a mild ARCS phenotype with prolonged survival. Consequently, we believe that the molecular analysis of the *VPS33B* and *VIPAS39* should be considered in patients with normal gamma-glutamyl transferase cholestasis.

## Introduction

Arthrogryposis, renal dysfunction, and cholestasis syndrome (ARCS) is a rare autosomal recessive multisystem disorder that has been named because of its three cardinal features (Nezelof et al., 1979; Horslen et al., 1994). There are two forms of the disease, with similar clinical symptoms: ARCS type 1 (ARCS1, OMIM 208085) is caused by pathogenic variants in the vacuolar protein sorting 33 homolog B (*VPS33B* gene; OMIM 608552), whereas ARCS type 2 (ARCS2, OMIM 613404) is caused by pathogenic variants in the VPS33B-interacting protein apical-basolateral polarity regulator spe-39 homolog (*VIPAS39* gene, also known as *VIPAR*, OMIM 613401). Germline variants in the *VPS33B* gene have been found in approximately 75% of patients with ARCS (Gissen et al., 2006).

Consistent with the widespread organ dysfunction in ARCS, VPS33B has a role in the regulation of intracellular protein trafficking, particularly with abnormal organelle biogenesis on the liver and on the kidney that may ultimately result in cholestasis and tubular disfunction (Gissen et al., 2004). VPS33B interacts with soluble N-ethylmaleimide–sensitive factor attachment protein receptors (SNAREs), which are involved in synaptic vesicle fusion, vesicular exocytosis, and neurosecretion (Lobingier and Merz, 2012; Han et al., 2017). In addition, mouse knockout studies have shown that *VPS33B* and *VIPAS39* are essential for epidermal lamellar body biogenesis and function (Rogerson and Gissen, 2018).

Clinical presentation, together with *VPS33B* and *VIPAS39* sequencing analysis, has been considered the recommended diagnostic procedure (Gissen et al., 2006; Cullinane et al., 2009; Zhou and Zhang, 2014). Organ biopsy, which was used in the past in combination with clinical diagnosis, has largely been replaced by molecular analysis, because more than 50% of patients are vulnerable to coagulation defects and kidney and/or liver biopsies may result in risk of fatal hemorrhage (Gissen et al., 2006; Zhou and Zhang, 2014). Nine of 11 patients that were reported with bleeding episodes had normal platelet morphology and count (Gissen et al., 2006). Clinical diagnosis of ARCS consists on identifying the triad conditions of arthrogryposis, renal tubular acidosis, and neonatal cholestatic jaundice with normal gamma-glutamyl transferase (GGT) activity (Gissen et al., 2006). No specific treatment currently exists for this syndrome. Rather, supportive care should be administered to patients with the aim of improving the quality of life (Zhou and Zhang, 2014). As additional features have been described, it has become evident that the phenotype is variable. For instance, renal disease can range from renal tubular acidosis to Fanconi syndrome or nephrogenic diabetes insipidus (Del Brio Castillo et al., 2019).

Comprehensive reviews have analyzed the clinical phenotype of more than 62 patients with ARCS1 and showed that the three cardinal features are sometimes accompanied by other phenotypic features, including ichthyosis, mild dysmorphic signs, platelet anomalies, agenesis of the corpus callosum, hypotonia, structural cardiac defects, deafness, recurrent infection, and severe failure to thrive (Abu-Sa’da et al., 2005; Gissen et al., 2006; Zhou and Zhang, 2014). Most patients have failed to survive beyond the first year of life because of recurrent infections, acidosis, or severe hemorrhage (Gissen et al., 2006; Zhou and Zhang, 2014). However, recently, some reported patients with milder phenotypes survived infancy, including cases with isolated liver disease (Agawu et al., 2019; Qiu et al., 2019).

Here, we report three patients with variants in *VPS33B* identified by whole exome sequencing (WES). They presented a mild ARCS phenotype with prolonged survival.

## Case Report

### Family 1—Patients 1 and 2

**FIGURE 1:**
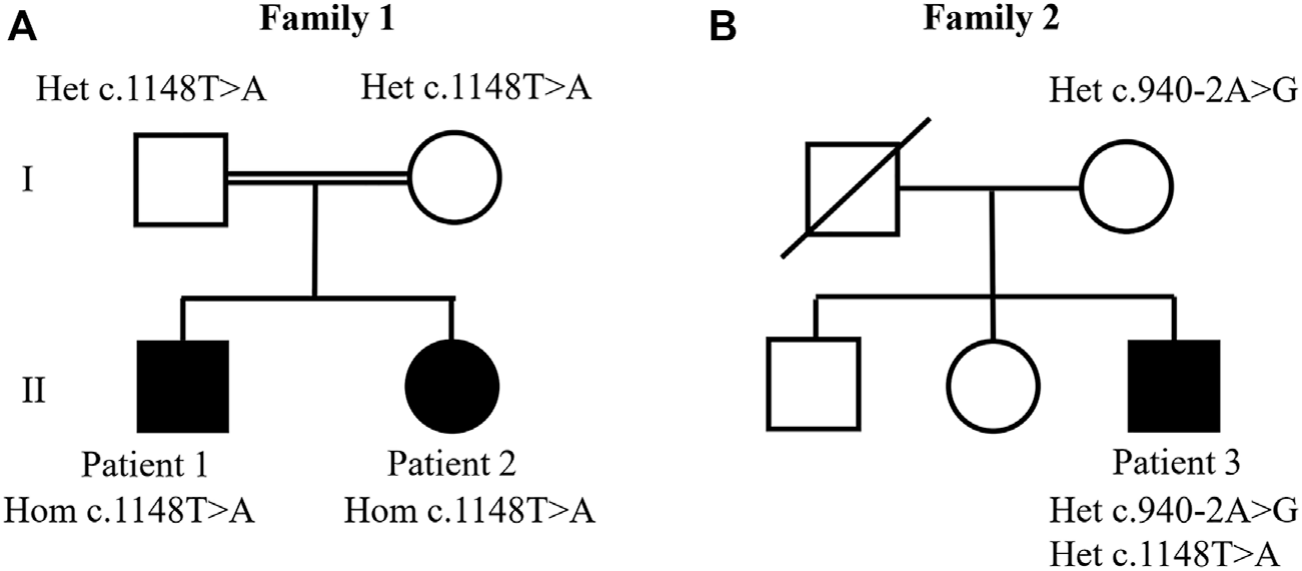
Studied families’ pedigrees. **(A)** Family 1: whole exome sequencing analysis identified the homozygous c.1148T>A. p.(Ile383Asn) variant in patients 1 and 2. **(B)** Family 2: compound heterozygous variants were identified in patient 3 at positions c.1148T>A. p.(Ile383Asn) and c. 940-2A>G.

Patients 1 and 2 were siblings born from a consanguineous healthy couple (their parent’s grandparents were siblings) (Figure 1). Patient 1 is a boy who was born at term with weight of 2,850 g (10th centile). Jaundice was noticed on first month and resolved spontaneously without additional inquiry. He started to have intermittent pruritus at 8 months of age. Neurodevelopmental delay was noticed at the second year of life. He acquired independent walking at 1 year and 6 months. He was evaluated with a clinical report of syndromic face, mainly characterized by the lack of hair on the eyebrows, low-set ears, discrete ptosis, discrete camptodactyly of fingers, tissue excess in the hands, hyperreflexia in the lower limbs, bilateral short hallux, gait with *equinovarus* on the left, and dry and scaling skin. Brain magnetic resonance imaging showed hypoplasia of the corpus callosum and dysgenesis in a small area of the left cerebellar hemisphere, suggesting an abnormality of neuronal migration. There was no growth delay. He was first seen at the Clínica de Hepatologia Pediátrica of Hospital das Clínicas of the Universidade Federal de Minas Gerais (UFMG) at age 9 due to severe and difficult control pruritus, which was persistent despite topic skin care and use of ursodeoxycholic acid and rifampicin. He had mild elevated aminotransferases with normal GGT. Bilirubin, albumin, prothrombin time, and partial thromboplastin time were normal. Other causes of liver disease, such as Wilson’s disease, hepatitis B and C, α1-antitrypsin deficiency, and autoimmune hepatitis, were excluded by laboratory tests. Liver biopsy showed preserved lobular architecture and mild portal mononuclear inflammatory infiltrate without fibrosis and cholestasis. He had normal platelet number and function without bleeding episodes and no renal dysfunction. He was studied at the Laboratório de Genômica Clínica at age 11. He was also referred to the Departamento de Neurologia Pediátrica, where he was followed due to neurodevelopmental delay, right hemiplegia, cognitive impairment, and behavioral abnormalities. Currently, at age 17, he maintains the cognitive and language delay.

Patient 2 is a girl who was born at term by normal delivery with weight 3,600 g (75th centile). However, she had low weight gain after 6 months of life. She presented jaundice, with spontaneous resolution and pruritus, although receiving ursodeoxycholic acid and rifampicin. She had mild elevated aminotransferases with normal GGT, hepatomegaly and decreased body weight and height, and normal liver function. She also presented facial dysmorphisms, dry and scaling skin, and neurodevelopmental delay with slow language development. She had no bleeding episodes and no renal dysfunction. Currently, at age 10, she presents mild pruritus, short stature, and normal liver and renal function.

### Family 2—Patient 3

Patient 3 is a boy who was born from non-consanguineous parents (Figure 1) and had healthy older brother and sister. He was born at term by cesarean delivery without complications with weight 2.910 g (10th centile). Intermittent jaundice started with 15 days of life. His mother noticed pruritus in the first months of life. He was followed at the Clínica de Hepatologia Pediátrica of Hospital das Clínicas of UFMG from the age of 4 years old. He presented cholestasis with intense pruritus, elevated aminotransferases, normal GGT, dry and scaling skin, developmental and language delay, sensorineural hearing loss, and syndromic facies characterized by low-set ears, broad forehead, brachycephaly, and short nasolabial filter, but he had no joint contractures. Other causes of cholestasis were excluded. Albumin, prothrombin time, and partial thromboplastin time were normal. Blood tests showed large platelet, however in normal number. He had no hemorrhage episodes. There was no renal dysfunction. Currently, at age 11, he presents mild pruritus and jaundice (total bilirubin, 5.6 mg/dl; direct bilirubin, 4.2 mg/dl) but normal liver function (normal albumin and prothrombin and partial thromboplastin time). He has normal growth but keeps developmental and language delay.

## Methods

### Samples, DNA Isolation, and WES Analysis

The Research Ethics Committee of the Hospital das Clínicas of UFMG approved the study protocol. Informed consent was obtained according to current ethical and legal guidelines. The study was conducted in accordance with the Declaration of Helsinki.

Genomic DNA was isolated from whole peripheral blood using a modified salting out procedure (Miller et al., 1988). It was not possible to collect patient 3’s father sample, because he was deceased.

WES was performed using patient 2’ sample by the Centre for Applied Genomics, Hospital for Sick Children, Toronto, Canada, using the SureSelect Human All Exon kit V5 (Agilent Technologies, Santa Clara, CA, USA), which targeted 21,522 genes and 357,999 exons, with a total size of 50 Mb. Enriched genomic DNA was sequenced on a HiSeq 2,500 Sequencer (Illumina, San Diego, CA, USA). The average coverage was 119,71X, with circa 95% of the target bases being covered at least at 20X.

WES of patient 3 was performed by Theragen Etex, Seoul, South Korea, using the SureSelect Human All Exon kit V6 (Agilent Technologies, Santa Clara, CA, USA), with a total target size of 58 Mb. Enriched genomic DNA was massively parallel sequenced on HiSeq 2,500 Sequencer (Illumina, San Diego, CA, USA). The average coverage was higher than 60X, with circa 70.9% of the target bases being covered at least at 20X.

All data were aligned to the GRCh37/hg19 reference genome build using the Burrows–Wheeler Aligner (BWA) aligner. Variants were called and quality trimmed using Genome Analysis Toolkit (GATK), and they were annotated for functional effect by SnpEff (Cingolani et al., 2012). Variants were filtered for rare variants (allele frequency < 0.005) utilizing databases such as 1,000 Genomes phase 3, NHLBI Exome Sequencing Project (ESP6500), Single Nucleotide Polymorphism database (dbSNP141), and gnomAD database using the Mendel, MD software developed in-house (Cardenas et al., 2017) and the ENLIS Genome Research software (Enlis Genomics, Berkeley, CA, USA). Only variants with impact moderate or high according to SNPeff were taken into account (Cingolani et al., 2012). To analyze the impact of the candidate variants, the software Alamut Visual version 2.15.0 (Interactive Biosoftware) was used, which showed the alignment of orthologous genes, displayed protein domains information from InterPro, and hosted protein function prediction tools such as SIFT, PolyPhen-2, MutationTaster, and Align GVGD (Tavtigian et al., 2006; Adzhubei et al., 2010; Schwarz et al., 2010; Sim et al., 2012). CADD and REVEL scores were also evaluated (Ioannidis et al., 2016; Rentzsch et al., 2019). Splice site predictions were performed using tools on Alamut Visual: MaxEntScan, SpliceSiteFinder-like, and NNSPLICE (Reese et al., 1997; Zhang, 1998; Yeo and Burge, 2004). Because the probands were Brazilians, the allele frequencies of the candidate variants were also investigated on the Online Archive of Brazilian Mutations (ABraOM), a repository containing genomic variants from 1,171 unrelated Brazilian individuals (Naslavsky et al., 2020).

### Sanger Sequencing

Sanger sequencing was performed for validation of the variants of interest identified by exome analysis using the BigDye Terminator v3.1 Cycle Sequencing Kit (Applied Biosystems) and the Applied Biosystems (ABI) 3,130 Genetic Analyzer. Sequencing data were analyzed using the software Sequencher version 4.1.4 (Gene Codes Corporation).

## Results

We evaluated different inheritance models to filter the variants detected by WES. The fact that all parents were healthy and that one family had two children affected rendered dominant inheritance (autosomal or X-linked) unlikely. In addition, X-linked recessive inheritance was unlikely, as family 2 had one affected female proband. Consequently, we tested the autosomal recessive model of inheritance, which received support from the presence of distant consanguinity on family 1. It resulted in the identification of *VPS33B* variants in all patients.

**FIGURE 2:**
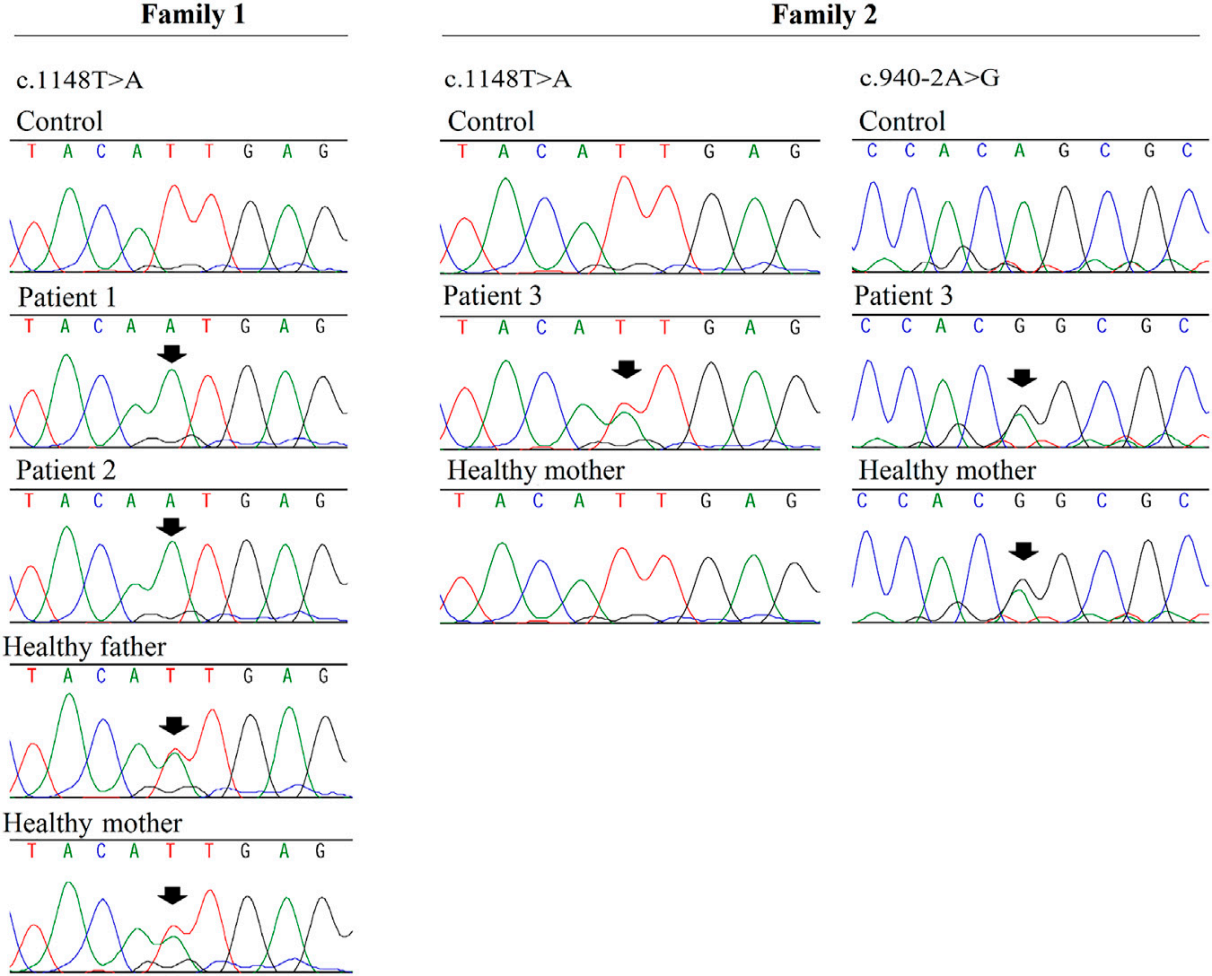
Fragments of Sanger sequencing chromatograms are shown for *VPS33B* gene. The black arrows indicate the variants positions. Patients 1 and 2 (siblings) are homozygous for the c.1148T>A variant and their parents are heterozygous; patient 3 is compound heterozygous for variants c.1148T>A and c.9 40-2A>G (which was inherited from the proband’s mother). DNA sample from patient 3’s father was not available.

Analysis of patients 1 and 2 identified a novel homozygous variant in exon 15 of *VPS33B*, at position chr15 (GRCh37):g.91548307A>T, NM_018,668.5(VPS33B):c.1148T>A. p.(Ile383Asn). The c.1148T>A. variant was classified as likely pathogenic (scores PM1, PM2, PM3, and PP3) according to the American College of Medical Genetics and Genomics (ACMG) and Association for Clinical Genomic Science (ACGS) Best Practice Guidelines (Richards et al., 2015; Ellard et al., 2020). This variant has not been previously described in patients with ARCS1 or in healthy individuals from worldwide populations according to the gnomAD database (Karczewski et al., 2019) and the ABraOM database (Naslavsky et al., 2020). The c.1148T>C variant (same position, but different nucleotide) has been previously registered in dbSNP under the code rs149121639, and it has been reported as having “uncertain significance” in ClinVar (RCV000372554.2). According to Alamut Visual software, the c.1148T>A. variant is located in well-established functional domains (Sec1-like, domain 2, and Vacuolar protein sorting-associated protein 33, domain 3b), and it has *in silico* pathogenic characteristics as assessed by the prediction programs SIFT (“deleterious”; score = 0.00), PolyPhen-2 (“probably damaging”; score = 0.995), MutationTaster (“disease causing”; p-value = 1), Align GVGD (Class C0), CADD (“deleterious”; score = 27.5), and REVEL (“deleterious”; score = 0.815). Altogether, the PM1 score was assigned because the variant is located in a well-established functional domain, PM2 was assigned because the variant was novel, PM3 was assigned because the variant was detected *in trans* with a pathogenic variant in patient 3, and PP3 was assigned because the variant was predicted to be pathogenic by computational tools. Sanger sequencing validated the homozygous variant in patients 1 and 2 and showed that their parents were heterozygous (Figure 2).

Compound heterozygous variants were identified in patient 3: he shared the same c.1148T>A. variant also present in patients 1 and 2, and additionally, he had a splice site variant located in the acceptor splice site of intron 12 of *VPS33B*, at position chr15 (GRCh37):g.91549016T>C, NM_018,668.5(VPS33B):c.940-2A>G. (HGMD accession CS041133) (Xiong et al., 2015). A skip of exon 13 was very likely; the predicted change at the acceptor site 2 bps downstream was 100% according to all splicing predictors shown on Alamut Visual (PVS1 score). This variant has been previously registered in the dbSNP dataset under the number rs774529051. Its allelic frequency was 0.00040% in healthy worldwide populations according to gnomAD database v2.1.1. This variant was classified as pathogenic according to ACMG and ACGS Best Practice Guidelines (scores PVS1, PM2, and PS4_Moderate). According to the gnomAD database, it has been reported in only one heterozygous individual from African population (PM2 score). This variant is absent from ABraOM database. It has been classified as “pathogenic” in the ClinVar database (accession RCV000730889.1), and it has previously been reported in one French individual (Gissen et al., 2006). Consequently, a PS4_Moderate score was assigned. Sanger sequencing showed that only the c.940-2A>G. variant was inherited from the mother (Figure 2). Thus, the c.1148T>A. variant could have been inherited from the deceased father, or it could have occurred *de novo* in the patient.

## Discussion

We described here three patients with mild phenotype of ARCS with *VPS33B* variants. Patients 1 and 2, who are siblings, presented a novel homozygous c.1148T>A. p.(Ile383Asn) variant, and patient 3 was compound heterozygous for the same c.1148T>A. variant and c.940-2A>G. variant.

To date, a total of 49 unique pathogenic *VPS33B* variants and 14 pathogenic *VIPAS39* variants are listed in the Leiden Open-Source Variation Database (LOVD) for ARCS (Smith et al., 2012; Zhou and Zhang, 2014). The variant distribution is relatively uniform within *VPS33B*, with no obvious mutational hotspots (Smith et al., 2012). The c.940-2A>G. variant, present in patient 3, has been reported in one French individual (pedigree 22); it was described in compound heterozygosity with a c.240-13delTT variant in the 2 months old proband who had cholestasis with normal GGT, renal Fanconi syndrome, arthrogryposis multiplex congenita, and failure to thrive (Gissen et al., 2006).

The phenotype of ARCS is compatible with the symptoms shown by our patients: all presented cholestasis, patient 1 had discrete camptodactyly of fingers, march with *equinovarus* on the left and suggestion of dysgenesis of the corpus callosum (Table 1). Gissen et al. (2006) analyzed the phenotype of 62 patients with ARCS and, similarly, to our patient 1, dysgenesis of the corpus callosum and other intracranial abnormalities were reported in nine patients. In addition, arthrogryposis was present in 60 patients, and its severity ranged from isolated talipes to congenital hip dysplasia (Gissen et al., 2006).

**TABLE 1:** Summarized clinical characteristics of the patients described here, and comparison with ARCS reported clinical features.

	Reported clinical features Gissen et al. (2006)	Patient 1	Patient 2	Patient 3
Nucleotide alterations	—	c.1148T>A.	c.1148T>A.	c.1148T>A. c.940-2A>G.
Alterations in coding sequence	—	p.(Ile383Asn)	p.(Ile383Asn)	p.(Ile383Asn) p.?
Zygosity	—	Hom	Hom	Het Het
Gender	—	Male	Female	Male
Age	—	17 years	10 years	11 years
Classical clinical features	—	—	—	—
Congenital joint contractures-Arthrogryposis	+	+ Mild flexion contractures	−	−
Renal tubular dysfunction	+	−	−	−
Cholestasis with normal GGT	+	+ Neonatal cholestatic jaundice	+ Neonatal cholestatic jaundice	+ Neonatal cholestatic jaundice
Additional clinical features	—	—	—	—
Failure to thrive	+	−	+ Short stature	−
Neurodevelopmental delay	+	+	+	+
Dysmorphic features	+	+	+	+
Hypotonia	+	−	−	−
Diarrhea	+	−	−	−
Cardiovascular anomalies	+	−	−	−
Ichthyosis	+	Dry and scaling skin	Dry and scaling skin	Dry and scaling skin
Recurrent sepsis	+	−	−	−
Dysgenesis of the corpus callosum	+	+	−	−
Sensorial hearing loss	+	−	−	+
Platelet alteration	+	−	−	+ Large platelet
Hemorrhage	+	−	−	−
Hypothyroidism	+	−	−	−

+, present; −, absent.

All patients analyzed by Gissen et al. (2006) presented difficulties in gaining weight and most of the patients died within the first 6 months of life. Possibly, due to an investigation bias, the diagnosis of ARCS only would be suggested by a severe phenotype with the three cardinal signals and then confirmed by molecular analysis of the *VPS33B* and *VIPAS39* genes.

The patients studied here shared the novel missense c.1148T>A. p.(Ile383Asn) variant and presented a mild phenotype of the ARCS, with cholestasis as a main feature and without arthrogryposis or renal dysfunction. Moreover, these patients are surviving much longer than the ones with typical ARCS phenotype. Currently, they have 17, 10, and 11 years old, respectively, and to our knowledge, patient 1 is one of the oldest patients described with ARCS to date. Other similar patients with milder phenotypes have been reported indicating the possibility of incomplete ARCS phenotype (Bull et al., 2006; Smith et al., 2012; Agawu et al., 2019; Del Brio Castillo et al., 2019; Qiu et al., 2019; Agakidou et al., 2020).

We then hypothesized that perhaps patients with missense variants in *VPS33B* gene might have an attenuated incomplete phenotype when compared with the ones with loss-of-function variants. On Supplementary Table S1, we compared our patient’s phenotype with other patients reported in the literature with missense variants (Gissen et al., 2004; Cullinane et al., 2009; Tornieri et al., 2013; Gruber et al., 2017; Del Brio Castillo et al., 2019; Lee et al., 2019; Qiu et al., 2019; Seidl-Philipp et al., 2020).

Only 11 patients have been reported with pathogenic *VPS33B* missense variants in the literature and two of them had no detailed phenotypic data described (Gissen et al., 2004; Cullinane et al., 2009; Tornieri et al., 2013) (Supplementary Table S1). With the exception of the patient described by Lee et al. (2019), all patients that were reported with missense variants had a milder phenotype. However, further patients with missense variants are needed to validate this hypothesis. The proband described by Lee et al. (2019) had a missense variant in one allele and a splice site variant in the other allele, which could be influencing her phenotype (she carried p.Asp236Val and c.239+5G>A variants). Interestingly, three patients with the same missense p.(Gly131Glu) variant were described as having the phenotype of Keratoderma-ichthyosis-deafness (ARKID) syndrome, a rare multisystem disorder also caused by biallelic mutations in *VPS33B* (Gruber et al., 2017; Seidl-Philipp et al., 2020). It is important to note that at least six patients with incomplete phenotype have been reported with loss of functions variants, which shows that an incomplete phenotype is not always caused by missense variants—these patient’s phenotypes were also summarized on the Supplementary Table S1 (Bull et al., 2006; Smith et al., 2012; Agawu et al., 2019; Agakidou et al., 2020; Duong et al., 2020b; a).

The genetic and clinical features of the previously reported patients were reviewed by Smith et al. (2012), and they provided the first evidence of genotype-phenotype correlation in ARCS. They reported two patients with an attenuated ARCS phenotype, who were compound heterozygous for the same c.1225+5G>C variant, resulting in the expression of a shorter VPS33B protein product that retained some ability to interact with VIPAS39. Other previous studies also suggested that variants in patients with complete ARCS phenotype caused absent VPS33B protein expression or abolished the interaction with VIPAS39, whereas variants in patients with attenuated phenotype would be less severe with partially preserved protein expression and function (Cullinane et al., 2009; Smith et al., 2012; Qiu et al., 2019).

Here, we report three patients with the same novel c.1148T>A. variant, and we believe that this variant could be associated with a mild phenotype. Further cell-based assays would be necessary to analyze if this variant would retain the VPS33B protein ability to interact with VIPAS39, similarly to the previously discussed studies (Cullinane et al., 2009; Smith et al., 2012; Qiu et al., 2019).

Those subtypes/incomplete phenotypes make it difficult to differentiate through routine clinical investigations without genetic analysis. Our patients with low GGT cholestasis would remain undiagnosed without genetic tests. Thus, we raised awareness of the mild clinical picture of ARCS, and we propose that molecular analysis of the *VPS33B* and *VIPAS39* should be considered in patients with normal GGT cholestasis, and not only for patients with the complete ARCS phenotype. Other potential cause of normal GGT cholestasis that should be considered is progressive familial intrahepatic cholestasis (PFIC), which is a heterogeneous group of autosomal recessive disorders that accounts for 10%–15% of the cholestasis cases in children (Davit-Spraul et al., 2009). Because PFIC has a higher incidence than ARCS, it should be initially considered. As a consequence of this approach, it is expected that the number of atypical ARCS diagnoses may increase as well.

## Conclusion

In conclusion, we described here three patients with ARCS diagnosed by WES analysis. They carry the same novel c.1148T>A. variant, and we believe that this variant could be associated with a mild phenotype. Classical clinical diagnosis would not be appropriate for patients with mild phenotype of this syndrome. We propose here that *VPS33B* and *VIPAS39* mutation screening in patients with normal GGT cholestasis could facilitate accurate diagnosis and the administration of supportive care at early stage, in addition to provide genetic counseling for the affected families. No specific treatment currently exists for ARCS, but advances in knowledge or ARCS pathogenesis may lead to novel therapies and improved management, which are valuable in patients with prolonged survival.

## Data Availability

The datasets for this article are not publicly available due to concerns regarding participant/patient anonymity. Requests to access the datasets should be directed to the corresponding author.
